# Bioinformatics Meets Virology: The European Virus Bioinformatics Center’s Second Annual Meeting

**DOI:** 10.3390/v10050256

**Published:** 2018-05-14

**Authors:** Bashar Ibrahim, Ksenia Arkhipova, Arno C. Andeweg, Susana Posada-Céspedes, François Enault, Arthur Gruber, Eugene V. Koonin, Anne Kupczok, Philippe Lemey, Alice C. McHardy, Dino P. McMahon, Brett E. Pickett, David L. Robertson, Richard H. Scheuermann, Alexandra Zhernakova, Mark P. Zwart, Alexander Schönhuth, Bas E. Dutilh, Manja Marz

**Affiliations:** 1 European Virus Bioinformatics Center, 07743 Jena, Germany; bashar.ibrahim@uni-jena.de (B.I.); a.andeweg@erasmusmc.nl (A.C.A.); akupczok@ifam.uni-kiel.de (A.K.); philippe.Lemey@rega.kuleuven.be (P.L.); Alice.McHardy@helmholtz-hzi.de (A.C.M.); dino-peter.mcmahon@bam.de (D.P.M.); bpickett@jcvi.org (B.E.P.); David.L.Robertson@glasgow.ac.uk (D.L.R.); RScheuermann@jcvi.org (R.H.S.); A.Schoenhuth@cwi.nl (A.S.); 2 Faculty of Mathematics and Computer Science, Friedrich Schiller University Jena, 07743 Jena, Germany; 3 Theoretical Biology and Bioinformatics, Utrecht University, 3508 TC Utrecht, The Netherlands; arkhipova.a.ksenia@gmail.com; 4 Department of Viroscience, Erasmus Medical Center, 3015 GD Rotterdam, The Netherlands; 5 Department of Biosystems Science and Engineering, ETH Zürich, 4058 Basel, Switzerland; susana.posada@bsse.ethz.ch; 6 SIB Swiss Institute of Bioinformatics, 4058 Basel, Switzerland; 7 Université Clermont Auvergne, CNRS, LMGE, F-63000 Clermont-Ferrand, France; Francois.ENAULT@uca.fr; 8 Department of Parasitology, Institute of Biomedical Sciences, University of São Paulo, 05508-000 São Paulo, Brazil; argruber@usp.br; 9 National Center for Biotechnology Information, NLM, National Institutes of Health, Bethesda, MD 20894, USA; koonin@ncbi.nlm.nih.gov; 10 Institute of General Microbiology, Kiel University, 24118 Kiel, Germany; 11 Clinical and Epidemiological Virology, Rega Institute, KU Leuven, University of Leuven, 3000 Leuven, Belgium; 12 Department for Computational Biology of Infection Research, Helmholtz Center for Infection Research, 38124 Braunschweig, Germany; 13 Institute of Biology, Free University Berlin, Schwendenerstr. 1, 14195 Berlin, Germany; 14 Department for Materials and Environment, BAM Federal Institute for Materials Research and Testing, Unter den Eichen 87, 12205 Berlin, Germany; 15 J. Craig Venter Institute, Rockville, MD 20850, USA; 16 MRC-University of Glasgow Centre for Virus Research, Garscube Campus, Glasgow G61 1QH, UK; 17 J. Craig Venter Institute, La Jolla, CA 92037, USA; 18 Department of Genetics, University Medical Center Groningen, 9700 RB Groningen, The Netherlands; sashazhernakova@gmail.com; 19 Department of Microbial Ecology, Netherlands Institute of Ecology (NIOO-KNAW), 6708 PB Wageningen, The Netherlands; m.zwart@nioo.knaw.nl; 20 Centrum Wiskunde & Informatica, Science Park 123, 1098 XG Amsterdam, The Netherlands; 21 Leibniz Institute for Age Research-Fritz Lipmann Institute, 07745 Jena, Germany

**Keywords:** bioinformatics, virology, viruses, software

## Abstract

The Second Annual Meeting of the European Virus Bioinformatics Center (EVBC), held in Utrecht, Netherlands, focused on computational approaches in virology, with topics including (but not limited to) virus discovery, diagnostics, (meta-)genomics, modeling, epidemiology, molecular structure, evolution, and viral ecology. The goals of the Second Annual Meeting were threefold: (i) to bring together virologists and bioinformaticians from across the academic, industrial, professional, and training sectors to share best practice; (ii) to provide a meaningful and interactive scientific environment to promote discussion and collaboration between students, postdoctoral fellows, and both new and established investigators; (iii) to inspire and suggest new research directions and questions. Approximately 120 researchers from around the world attended the Second Annual Meeting of the EVBC this year, including 15 renowned international speakers. This report presents an overview of new developments and novel research findings that emerged during the meeting.

## 1. Introduction

Bringing together the expertise of bioinformaticians and virologists is crucial, because often fundamental computational issues need to be discussed that emerge from specific questions arising in virus research. The issues are further accentuated by the fact that the era of big data has also reached virology, in full strength. The European Virus Bioinformatics Center (EVBC) aims at reinforcing the synergy between bioinformatics and virology at all levels, including national and international funding scales, which is an urgent issue [1,2]. Collaboration between virologists and bioinformaticians is necessary to establish improvements with respect to the adaptation of analytical tools to particular requirements in virology, the development of cloud-based systems, the general assessment of computational resources, and the understanding of how data can be shared efficiently. New diagnostic tools and training in bioinformatics for virologists are also in high demand. Establishing these improvements requires concerted efforts, across country borders. The Second Annual Meeting of the EVBC was held at Utrecht University, Netherlands on 9–10 April 2018, in response to these issues, and to discuss new advances and solutions. The Second Annual Meeting followed up on the successful first meeting of the EVBC in Jena on 6–8 March 2017, so as to preserve the current momentum and point out that the EVBC intends to establish a series of meetings to be held on a regular basis. The Scientific Committee places high expectations on the excellence of the scientific program, which includes invited lectures from renowned scientists whose work has been an inspiration for researchers in virology and bioinformatics. The meeting allowed many interactions to take place between young and senior scientists from academia as well as from the private sector. Over the two days, the meeting was characterized by presentations to broadly cover topics from the field of virus bioinformatics. These presentations are summarized in this report. The invited speakers presented very interesting talks and stimulated interactive discussions that continued during coffee breaks and the ample poster sessions.

## 2. Selected Invited Lectures

A number of presentations were given by leading virologists and bioinformaticians; highlights included the following:

### 2.1. Eugene Koonin Presented a Keynote Lecture on the Expansion of the Virus World in the Era of Metaviromics

Metagenomics is changing the existing ideas on biodiversity and on the diversity and scope of the earth’s virome in particular. Already at this time, the number of new viruses that are being discovered by metagenomic approaches (metaviromics, for short) by far exceeds that identified by traditional approaches. Accordingly, the International Committee on Taxonomy of Viruses (ICTV) has adopted new rules that allow new virus species and higher taxa to be formally recognized on the basis of metagenomic sequence alone, provided that the respective genome(s) is (are) (nearly) complete and that adequate genome analysis is presented [3]. Metaviromics has led to the discovery of major new groups of viruses, the existence of which has not even been suspected previously. A striking example is the identification of an expansive family of bacteriophages that are associated with bacteria of the phylum Bacteroidetes and include crAssphage, the most abundant virus in the human virome [4,5]. Other discoveries in the same vein include the identification of the first family of viruses infecting Marine Group II archaea, a major group of uncultivated microbes [6], and a new group of giant viruses, Klosneuviruses, that encode a far larger repertoire of translation system components than any previous known viruses [7]. This latter finding is an illustration of the potential of metaviromics to uncover new biology, above and beyond the expansion of the virome diversity. Metaviromics is also changing the existing ideas on the evolution of viruses, in particular, by showing that the horizontal transfer of viruses between diverse hosts is far more common than previously thought and, to a large extent, shapes the evolution of the virus world [8].

It should be emphasized that, to take the full advantage of the rapid proliferation of metagenomic data, it is essential to apply the full armory of the most powerful and robust methods for protein-sequence and -structure analysis. In the years to come, metaviromics can be expected to advance at an accelerating rate and, combined with strategic choice of virus models for experimental study, will be the foundation of the new virology. One of the expected outcomes of the advances of metaviromics is a complete overhaul of virus taxonomy.

### 2.2. Anne Kupczok Talked about the Contribution of Mutation and Recombination to Dairy Phage Genome Evolution over Three Decades

Virus evolution is driven not only by the inheritance of genetic modification but also by the acquisition of foreign DNA. The evolutionary processes that affect genomic diversity include point mutation, recombination, and gene turnover. The temporal dynamics of these molecular events are crucial for our understanding of virus genome evolution. Heterochronous sequences, that is, sequences from isolates that were sampled at different time points, can provide detailed evidence for the evolutionary processes that shaped the populations over the sampling time. Evolutionary rates have been estimated for bacteria and for eukaryotic viruses but only rarely for bacteriophages. Phages are recognized as major contributors to bacterial evolution and ecology in nature. Thus, the estimation of phage evolutionary rates provides the basis for a quantitative assessment of phage evolutionary rates that are in action during phage bacteria antagonistic coevolution.

We recently estimated evolutionary rates from heterochronous dsDNA phage genomes [9]; 34 isolates of the 936 group of Siphoviridae phages were isolated using a *Lactococcus lactis* strain from a single dairy over 29 years. The analysis pipeline used was based on publicly available tools that were combined with the assessment of temporal signals at all stages of the analysis. Furthermore, it was necessary to disentangle vertical and horizontal evolutionary processes to detect a separate molecular clock signal in substitutions originating from mutational processes and in recombination events. Using this approach, we estimated substitution and recombination rates individually. The substitution rate of $1.9\times 10^{-4}$ substitutions per site per year estimated for lactococcal phages is within the range of estimates for eukaryotic dsDNA viruses (∼$10^{-5}$) and eukaryotic RNA viruses (∼$10^{-3}$) [10]. The reconstruction of recombination events revealed a rate of five recombination events per year and $4.5\times 10^{-3}$ nucleotide alterations due to recombination per site per year. Thus, the recombination rate exceeded the substitution rate, resulting in a relative effect of recombination to mutation (r/m) of ∼24, which was homogenous over time. The analysis of bacterial genome-wide data resulted in generally low r/m values, for example, 0.283 in *Staphylococcus aureus* [11] and 7.2 for a *Streptococcus pneumoniae* lineage isolated over 20 years [12]. Thus, on the basis of our estimate of r/m for a distinct Siphoviridae phage lineage, we conclude that the effect of recombination relative to mutation can be elevated in phage genome evolution in comparison to bacteria. Frequent gene loss and regain due to recombination with phages of the 936 group resulted in no detectable temporal signal in gene content variation. Variable protein families were enriched in the early transcriptional region that typically encodes non-essential proteins involved in phage–host interaction. The observed recombination events are best explained by host coinfection by related, yet genetically different, phages. The genetic connectivity within the pangenome indicates that different phages of the 936 group share an environment in which frequent coinfection events occur. Hence, different phage types can propagate and evolve in an undefined starter culture that comprises diverse host strains.

The analysis of heterochronous sequencing data requires appropriate mathematical models and bioinformatic tools. Models and tools for substitution rate estimation from heterochronous data are well established (e.g., [13]). It is advised that recombination is detected and recombinant lineages or sites are excluded before the substitution rate analysis [14]. Various methods and approaches are available for this task (e.g., [11,15]), and none of these methods have been designed for heterochronous data. Recent methodological developments for the inference of the bacterial recombination graph in the BEAST2 framework [16] are promising also for the inference of recombination in phages and viruses. To conclude, further development of mathematical models and bioinformatic tools for the joint estimation of substitution, recombination, and gene turnover rates from heterochronous data is necessary to provide an integrated framework for evolutionary rate estimation. These computational methods will enhance our understanding of the temporal dynamics of these processes and of virus genome evolution in general.

### 2.3. David Robertson Shared His Experience of Studying Virus–Host Interaction Networks and Modeling Viral Control of the Host System

David presented his research group and collaborator’s work on virus–host molecular interaction networks. Here, the focus was on the intracellular ”living” aspect of the virus, the exploitation of the host system. This directed the study of viruses on both their own and their host’s genome/proteome and the combined molecular interactions involved in the intracellular dynamics of infection. Most research in this area has been on HIV-1, as it is for this system that the majority of experimental work exists. This data was first painstakingly captured from the literature and visualized 10 years ago [17]. In particular, it has been found that viruses tend to target highly connected proteins, that is, hubs, in the host because of a combination of these being evolutionarily older molecules and thus more functionally ”central”, because they are more efficient for optimal control of the cell, and because of the over-representation of hubs in the main functions exploited by HIV-1 [18,19]. While these bipartite networks have been useful for representing viral infection, they are essentially a static representation of a dynamic system. Our aim has been to introduce a dynamic perspective by focusing on directed interactions in signaling networks. Logical models, networks that use logical boolean formalism to represent interactions, are one way of considering the directed interactions and permit in silico knockout experiments [20]. This approach enables the virus interactions to be explicitly linked to their downstream effect.

In more recent work, we have applied control theory to the virus–host interaction network [21]. A system is controllable if with a suitable choice of inputs we can ”drive” the network to a required state. The aim is to identify the driver nodes, the set of molecules that can control the network. Two states of a large signaling network were compared: uninfected/normal and infected with HIV-1. The driver nodes and a subset of critical driver nodes in the directed signaling network were identified before and after infection. Interestingly, the critical driver nodes had a tendency to be targeted by the virus, highlighting the way in which HIV-1 interacts with a complex ”moving” system. This emphasizes how a virus takes control over the infected cell in a highly orchestrated manner. Our model indicates that a virus fundamentally changes the controllability of the host system and uses an existing intracellular network to control the cell; that is, a virus drives the network by targeting existing critical driver nodes. As these are often hubs in the host network, this explains their over-representation in the set of molecules targeted by HIV-1. Control theory thus gives a more nuanced understanding of infection than previous approaches limited to static single-state networks.

### 2.4. Arno Andeweg Took Delegates through Probing the Host Response to Viral Infection Using Omics Approaches

To support the development of novel or improved antiviral strategies such as vaccines and biomarkers for personalized medicine, ”omics tools” are used to study the induction and regulation of the host (immune) response to viral infections. In a biomarker study, we investigated whether there exists a blood genomic signature that can accurately predict the course of respiratory syncytial virus (RSV) infection in young infants. RSV causes infections that range from the common cold to severe lower respiratory tract infection requiring high-level medical care. The prediction of the course of disease in individual patients is challenging at first visits to pediatric wards, as RSV infections may rapidly progress to severe disease. We used early blood microarray transcriptome profiles collected from 39 hospitalized infants (collected within 24 h of hospitalization) that were followed until recovery and for whom the level of disease severity was determined retrospectively. Applying support vector machine learning on age-by-sex standardized transcriptomic data, an 84-gene signature was identified that discriminated hospitalized infants with eventually less severe RSV infection from infants that suffered from the most severe RSV disease. This signature yielded an area under the receiver operating characteristic curve (AUC) of 0.966 using leave-one-out cross-validation on the experimental data and an AUC of 0.858 on an independent validation cohort consisting of 53 infants [22]. The presented signature may serve as the basis on which to develop a prognostic test to support clinical management of RSV patients. Follow-up studies are, however, needed to assess the pathogenic-specific—and/or disease-severity-specific—nature of the gene signature identified. Genome-wide association studies may identify host genetic factors predisposing to severe disease, but other approaches such as immune repertoire sequencing may help us to assess the contribution of other (external) factors, such as, for example, infection history to the development of (severe) disease upon infection. As a first step in this direction, we developed RTCR: a pipeline for complete and accurate recovery of T-cell repertoires from high-throughput sequencing data. This pipeline supports distinguishing between true but rare TCR sequences and variants generated by polymerase chain reaction and sequencing errors. RTCR employs a data-driven statistical model to rectify PCR and sequencing errors in an adaptive manner. Using simulations, we demonstrated that RTCR can easily adapt to the error profiles of different types of sequencers and exhibits a consistently high recall and high precision even at low coverages at which other pipelines perform poorly [23]. Using the RTCR pipeline, we analyzed a longitudinal dataset obtained from a healthy donor. The results showed that a healthy repertoire is highly dynamic: T-cell memory clone sizes appeared to undergo major shifts in abundance, demonstrating that small snapshots of the full repertoire nevertheless contained a wealth of information. Pattern analysis of paired (longitudinal) datasets will allow detailed studies of infection and, for example, vaccination-induced host responses.

### 2.5. Dino McMahon Gave a Powerful Talk on Evolution, Virulence, and the Recombination of Emerging Bee Viruses

**Figure 1:**
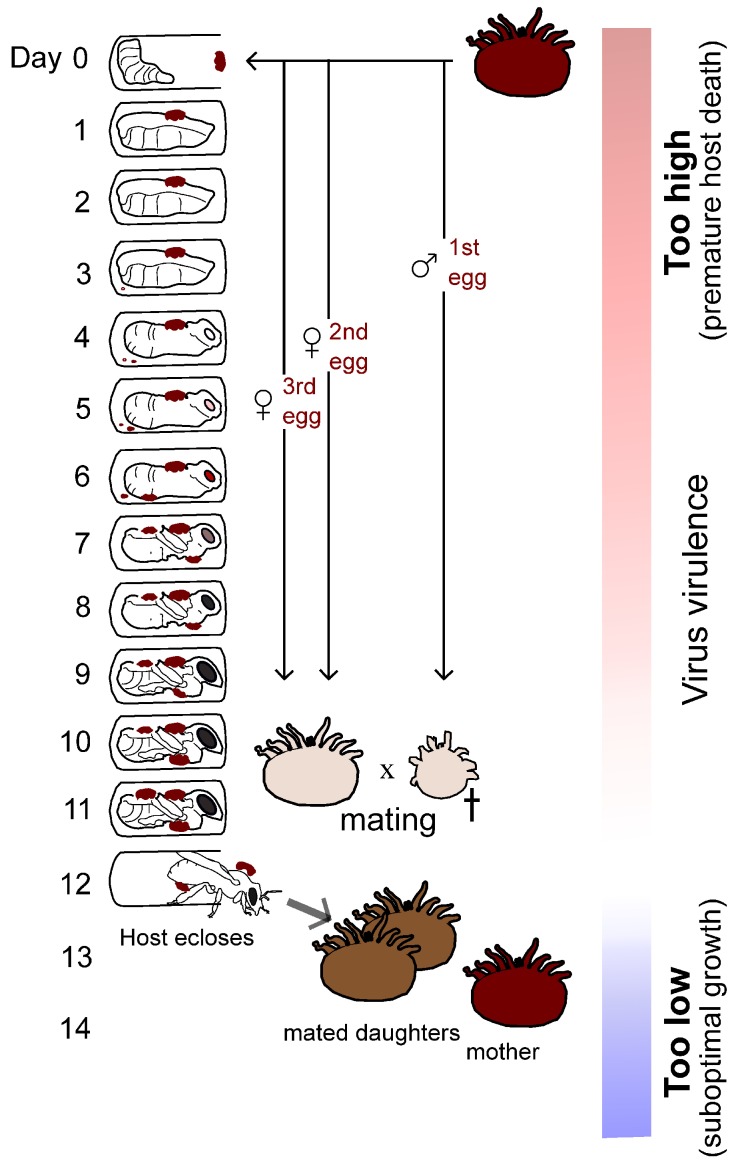
Hypothetical impact of indirect vector transmission on virus virulence evolution in *Apis mellifera*. The Varroa destructor reproductive life cycle is shown, with mites depicted at various developmental stages. A mother mite lays maximally three eggs that can mature to adulthood before hosts emerge from the brood cell. Virulence should evolve to an intermediate level that maximizes transmission (white region). Too virulent (red region) and the virus will have suboptimal transmission as a result of high host mortality preventing mite mating/host eclosion; not virulent enough (blue region) and the virus will have suboptimal transmission as a result of low growth (fewer transmission units). Reproduced with permission from McMahon, D.P.; Wilfert, L.; Paxton, R.J.; Brown, M.J.F. Emerging viruses in bees: from molecules to ecology. *Adv. Virus. Res.* **2018**, in press. (https://doi.org/10.1016/bs.aivir.2018.02.008).

Bee populations have declined significantly in recent years, and this is thought to be attributable at least in part to the (re-)emergence of viruses. These viruses are predominantly positive single-stranded (+ss) RNA viruses belonging to the Picornavirales. Managed honeybees are often infested with the invasive mite, *Varroa destructor*, which vectors RNA viruses, including deformed wing virus (DWV; family Iflaviridae), a leading culprit of colony losses. Many bee viruses have been sequenced, and structural features are now available for viruses such as DWV. DWV consists of at least three distinct genotypes, two of which have been shown to be differentially virulent in honeybees. Molecular studies have demonstrated that DWV has a mean evolutionary rate of 1.35 × $10^{-3}$ per site per year. For such viruses—in contrast with their eukaryotic hosts—ecological and evolutionary timescales significantly overlap. This rapid evolution allows RNA viruses to adapt quickly to novel host environments, with recombination representing an additional key source of genetic variation. Interestingly, recombination between genotypes of DWV has recently been shown to be a common occurrence in honeybees. A challenge has been to develop bioinformatics tools that can accurately reconstruct viral haplotypes—including recombinants—from heterogenous high-throughput sequence data. The impact of blood-to-blood *V. destructor* transmission on virus evolution represents an important question in bee virus research. Because of the nature of the *V. destructor* life cycle, predictions can be made about the potential impact of the mite on virus virulence evolution. Specifically, the developing honeybee host pupa should remain alive until close to the completion of metamorphosis to provide sufficient time for successful mite reproduction, including offspring mating. For optimal transmission, any virus found in a mature and mated daughter mite will hold a significant selective advantage over a virus found in an immature or unmated daughter mite—placing a cost on virus virulence that impacts honeybee pupae before mites can mate. On the other side, viruses replicating too slowly and with delayed virulence effects will hold a selective handicap because fewer transmission units will be found in mated mites. I have hypothesized that the evolution of virus virulence shifted following the arrival of *V. destructor*, with viruses, including recombinants and/or specific viral genotypes, being selected for a level of virulence in pupae (and likely also in adults) that maximizes R0, which represents the basic reproductive number of the virus in the host population. R0 is defined by the number of subsequent infections caused by a single infection, and it must be greater than 1 for an infection to spread in a population. It is enhanced by maximizing the number of transmission units passed to uninfected susceptible vectors and ultimately hosts (Figure 1).

Honeybee viruses are also shared with sympatric wild bees, and viral prevalence and sequence data indicate frequent virus transmission between managed and wild-bee species. In addition to infecting the western honeybee (*Apis mellifera*), DWV can infect other Asian honeybee species, such as *Apis ceranae*. Aside from honeybees, DWV has been found widely in bumblebees, including solitary bees, and in wasps, and there is evidence that it can actively replicate in several *Bombus* and solitary-bee species. Whether the arrival of the *V. destructor* mite in *A. mellifera* has driven the viral emergence in non-*Apis* bees is a target of ongoing research.

### 2.6. Arthur Gruber Talked about Rational Design of Profile HMMs for Viral Detection, Classification and Discovery

**Figure 2:**
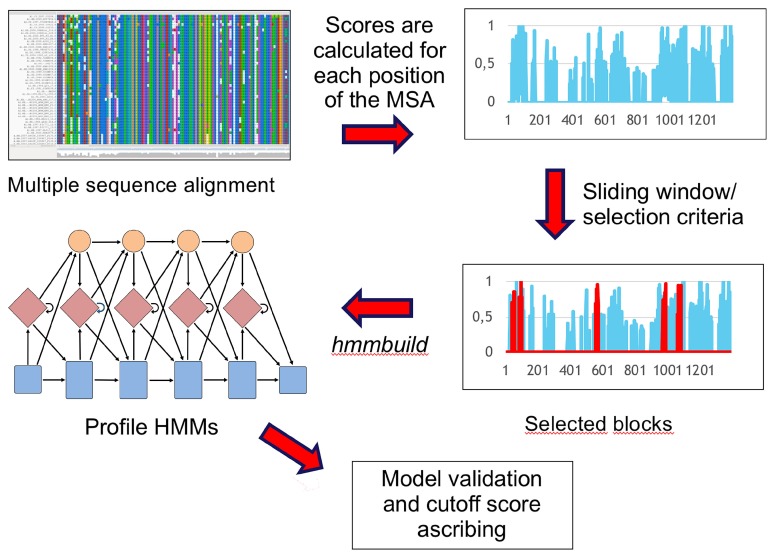
Workflow of TABAJARA program.

Some of the most devastating pandemic diseases have arisen through the transmission of emerging viruses that have not been identified before the tragic consequences of their dissemination. The detection of novel viruses is a challenging task because of their high evolutionary rates, which result in high sequence divergence. The development of new computational approaches is, therefore, of utmost importance. Profile HMMs are a powerful way of modeling sequence diversity and constitute a very sensitive approach to detecting emerging viruses [24]. Our group has recently developed GenSeed-HMM [25], a tool that employs profile HMMs as seeds for progressive assembly, using genomic and metagenomic data. Here we report the development and implementation of TABAJARA (Figure 2), a tool for the rational design of profile HMMs. Starting from a multiple sequence alignment (MSA), TABAJARA is able to find blocks that are either (1) conserved across all sequences or (2) discriminative for two or more specific groups of sequences. The program uses different metrics to ascribe position-specific scores along the whole alignment and then uses a sliding window to find top-scoring regions. Selected alignment blocks are then extracted and used to build profile HMMs. To validate such models, we used sequence data from phages of the Microviridae family and members of the *Flavivirus* genus. In both viral groups, we were able to obtain wide-range seeds (generic for all members of Microviridae or *Flavivirus*) and narrow-range seeds, exclusive to specific Microviridae subfamilies (Alpavirinae and Gokushovirinae) or to particular *Flavivirus* species (e.g., DENV, ZIKV, or YFV). Additionally, using TABAJARA’s generated profile HMMs as seeds, we succeeded to reconstruct specific viral sequences with the GenSeed-HMM program using different metagenomic datasets. Finally, we also report the development of e-Finder, a program that uses profile HMMs to identify clusters of genes in a specified syntenic context. This tool allows the detection of viral and nonviral elements such as prophages, transposons, CRISPR-Cas systems, and operons in assembled genomes. These programs constitute a toolbox and can be used alone or in combination to detect and classify known viruses as well as distantly related viruses that may represent potentially emerging pathogens. This work was done by the team composed of Liliane S. Oliveira, Dolores U. Mehnert, Paolo M. A. Zanotto, Alan M. Durham, Alejandro Reyes, and Arthur Gruber.

### 2.7. Richard Scheuermann Shared His Great Experience with Contemporary Circulating EV-D68 Strains Showing Differential Viral Entry and Replication in Human Neuronal Cell Cultures

Historically, enterovirus D68 (EV-D68) has only been associated with respiratory illnesses. However, in the summer of 2014, an EV-D68 outbreak coincided with a spike in polio-like acute flaccid myelitis/paralysis (AFM) cases. Statistical analysis suggested that the number of AFM cases was significantly higher during the EV-D68 outbreak than in historical controls. These reports have raised concerns that the EV-D68 virus could have been the causative agent of AFM during this recent outbreak. We recently reported a phylogenetic analysis of the 2014 outbreak and found that isolates associated with AFM belonged to a single phylogenetic subclade, B1 [26]. To determine if specific EV-D68 genetic determinants are associated with neuropathogenesis, we used a neuroblastoma-derived neuronal cell line, SH-SY5Y, as a cell culture model to explore the differential infection permissibility for different EV-D68 strains. In contrast to HeLa and A549 cells, which support viral infection of all EV-D68 strains tested, SH-SY5Y cells only support infection of a subset of contemporary EV-D68 strains, including MO/14-18947 and MO/14-18949, members of the B1 subclade from the 2014 outbreak. Viral replication and infectivity in SH-SY5Y was assessed using four independent assays—infectious virus production, cytopathic effects, cell viability assay, and VP1 capsid protein production. Similar differential neurotropisms were observed in a paralytic mouse model. In addition to supporting virus replication and other functional studies, this cell culture model may help to confirm epidemiological associations between EV-D68 strains and AFM and allow for the rapid identification of emerging neurotropic strains of EV-D68.

This work was done by David M. Brown, Lauren M. Oldfield, Alison M. Hixon, Yun Zhang, Mark Novotny, Wei Wang, Suman Das, Reed S. Shabman, Kenneth Tyler, and Richard H. Scheuermann. The study is funded by the National Institute of Allergy and Infectious Diseases (NIH/DHHS) under Contract No. HHSN272201200005C.

### 2.8. Alexandra Zhernakova Presented Virome Analysis Using Metagenomics Sequencing Data from Population and Patient Cohorts

**Figure 3:**
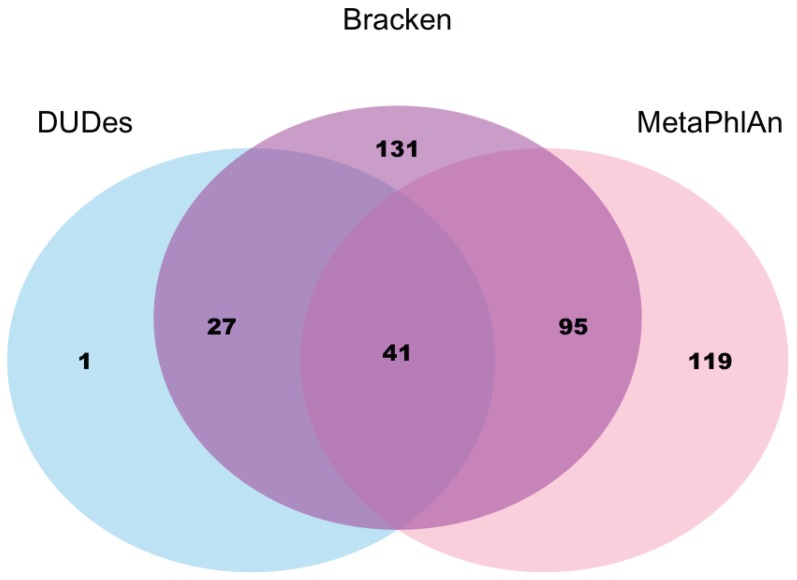
Number of shared samples with detected viruses (k_Viruses) in all three methods.

The human gut microbiome contains millions of viruses, bacteria, protozoa, and fungi. The abundance of bacterial species and the diversity of bacterial compositions have now been linked to many host diseases and conditions, in particular, to predispositions to autoimmune diseases, metabolic conditions, obesity, and cancer. Although the bacterial part of the human microbiome has been relatively well studied, the virome contribution remains largely unexplored, despite gut viruses forming a substantial part of the total microbiome: the proportion of viruses in adults is estimated to be 5–10% of the number of bacteria. Several pioneering studies have indicated that viruses are relatively stable in time and are associated with gut diseases, particularly with inflammatory bowel disease. Virome-specific isolation of nucleic acids from stool samples requires additional, currently laborious, methods. However, given the high abundance of viruses in the gut, metagenomic sequencing of all DNA isolated from stool should contain a substantial proportion of viral DNA. In this work, we aimed to investigate the gut virome abundance in a population cohort, Lifelines-DEEP (1179 samples), and compared it that of with patients suffering from inflammatory bowel disease (*n* = 355) and irritable bowel syndrome (*n* = 400). We tested three tools commonly used for the analysis of gut metagenomics data: (1) MetaPhlAn; (2) Kraken; and (3) DUDes. All three tools detected viruses in only a minority of the Lifelines-DEEP samples (25% in MetaPhlAn, 27% in Kraken, and 8% in DUDes), with moderate overlap across the three methods (Figure 3). The tools also indicated a low overall abundance of viruses (<1%) and a moderate overlap in taxa detected across virome-positive samples. The proportion of identified viruses did not correlate with the read depth of metagenomic sequencing. The most abundant viral family detected by all three methods was Siphoviridae, double-stranded DNA viruses that infect bacteria and archaea. This family contains several bacteriophages specific for *Lactococcus* and *Leuconostock* bacteria, and, consistently with this, we also observed a positive correlation of Siphoviridae viruses with the abundance of these bacteria and with the intake of milk products. We further observed an increased proportion of viral species in individuals with inflammatory bowel disease and irritable bowel syndrome. Overall, our virome analysis using existing metagenomics tools yielded unexpectedly low proportions of viruses in the stool samples of healthy individuals, and we saw remarkable differences in the results of the different tools. It is likely that virome reads remain in the unannotated fraction of the metagenomics data, which is substantial in all three methods (at least 30%). Other virome-specific tools and de novo data assembly methods are therefore required to determine the true viral proportion and abundance in metagenomics data. Data analysis for this project was performed by Sanzhima Garmaeva, PhD student in the Department of Genetics, UMCG.

### 2.9. François Enault Introduced the Study of Viral Communities Using Metagenomics

Although viruses are considered as major players in natural ecosystems, their comprehensive study has been hampered by the necessity of isolating and cultivating their host and by the absence of a single gene common to all viral genomes. Viral metagenomics, based on culture-independent sequencing, is a well-fitted approach to provide insights into the composition, structure, and dynamics of environmental viral communities. However, specific computational methods are required to analyze these viral metagenomes (or viromes). First, we developed a method to estimate the proportion of cellular sequences in viromes. A quarter of the published datasets were found to contain a substantial number of sequences originating from cellular genomes, either as a result of shortcomings in experimental protocols, as virus-like particles (VLPs) are described as difficult to purify from some matrices (soil, sediments, and animal-associated samples), or because of VLPs containing cellular DNA (membrane vesicles, gene transfer agents, or due to phage transduction). When considering viromes with no cellular DNA detected, a significant number of cellular metabolism genes were retrieved, for example, some ribosomal proteins, suggesting that the presence of auxiliary genes involved in various metabolic pathways within viral genomes is a general trend in the virosphere [27]. We then applied this method to quantify the presence of cellular DNA in gut viromes, as recent virome analyses suggest that antibiotic resistance genes (ARGs) are frequently carried by phages, which is inconsistent with the traditional view that phage genomes rarely encode ARGs but contribute to their spread through horizontal transfer mediated by generalized transduction. The reanalysis of available human- or mouse-associated gut viromes for ARGs and their genomic context suggested that ARGs are rarely encoded in phage genomes but more likely originate from phage particles containing bacterial DNA [28].

Considering the fact that viromes can contain a substantial number of sequences originating from cellular genomes, a tool was developed to identify viral sequences in such datasets. Using both homology to reference viral sequences and viral genome characteristics, VirSorter complements existing prophage prediction software and was proved to be able to identify viral signals in assembled sequence (contigs) as short as 3 kb [29].

Once the viral contigs are identified, determining the identity of their hosts is crucial for deciphering the interactions and dynamics between microbes and their viruses and thus for our ecological understanding of the microbiome. To determine the hosts of the newly sequenced viruses, we used the fact that viruses tend to have a genomic signature similar to their prokaryotic hosts. Using homogeneous Markov models, our tool WisH was found to predict hosts for short phage genomic fragments with a better accuracy than existing tools such as VirHostMatcher [30]. Furthermore, WisH runs several hundred times faster than this software and scales up to large datasets [31].

Using bioinformatic methods and software, we studied viral communities in aquatic systems such as freshwater lakes and hypersalin ponds, and in the context of the Virus-X EU project (Horizon 2020 Project No. 685778), we mostly focused on Icelandic hot springs. The analysis of viromes informs us about the composition, diversity, and dynamics of the viral communities in these environments and allowed us to analyze the main viral families retrieved in these datasets, mostly Caudovirales and ssDNA viruses such as Microviridae.

This work was done by Simon Roux, Clovis Galiez, Jonathan Vincent, Marie-Agnès Petit, Matthew B. Sullivan, Johannes Söding, Didier Debroas, Mart Krupovic, and François Enault.

### 2.10. Brett Pickett Discussed Genome Sequencing and Serodiagnostics for Zika Virus

Zika virus (ZIKV) is responsible for an ongoing outbreak concentrated in South and Central America and the Caribbean, with travel-based cases being reported around the globe. In addition, new outbreak clusters located in the southeastern United States have been attributed to autochthonous transmission. As multi-disciplinary collaborations are forming to combat the ZIKV outbreak, it is imperative to authenticate and sequence-verify existing reagents prior to performing infection experiments to better understand and combat increasing virulence. Here, we report high-quality sequence data for multiple ZIKV strains available from the Biodefense and Emerging Infections Research Resources Repository (BEI Resources). These sequence data include the historical reference strain from the African lineage (MR-766), the Asian lineage derived American reference strain (PRVABC59), and several other strains collected from around the world during the 2015 outbreak with documented passage histories. We used next-generation sequencing coupled with 3’ rapid amplification of cDNA ends (RACE) and viral genome annotation pipelines to generate consensus sequence records in GenBank. We applied a deep-sequencing analysis method to identify minor variants, which exist as natural quasi-species within viral stocks. We detected minor variants that consistently resulted in nonsynonymous substitutions at multiple codons among a subset of the Asian strains. Interestingly, the nonstructural protein 1 (NS1) and membrane glycoprotein (prM) coding regions had the highest number of minor variants in the Asian and African lineages, respectively. The evolutionary relationships for these strains were determined using phylogenetic methods, and a single recombinant sequence was identified. These bioinformatics analyses of the sequencing results confirm that the BEI Resources virus stocks available to the scientific research community adequately represent the viral population diversity of ZIKV and justify additional wet-lab experiments. In addition, we used peptide arrays consisting of 866 15-mer peptides (in quadruplicate) to screen 140 well-characterized human convalescent sera for peptides that differentiate between past infection with multiple mosquito-borne viruses. Peptides representing dengue virus serotypes 1–4 and West Nile, Chikungunya, and Zika viruses were synthesized chemically and linked covalently to glass slides prior to measuring peptide-specific binding of anti-virus IgG for these seven mosquito-borne viruses. The data from all arrays were combined prior to applying multiple rounds of machine learning, a weighting scheme, and a B-cell epitope prediction algorithm. Together, this novel computational workflow identified pools of 10 immunodominant 15-mer peptides for each of the viruses. An indirect ELISA protocol was optimized to enable the validation of our selected 15-mer peptides against large collections of clinical serum samples. These experiments will enable the quantification of the diversity in antibody responses against mosquito-borne viruses in human patients and can be used to enhance vaccine development efforts, improve point-of-care diagnostics, and enable more accurate calculation of seroprevalence in endemic areas.

### 2.11. Alice McHardy Presented the Prediction of the Evolution of Human Influenza A Viruses

Human influenza viruses cause short-term respiratory infections with considerable morbidity and mortality in seasonal epidemics. A trivalent or quadrivalent vaccine protects against the two circulating subtypes of influenza A (pH1N1 and H3N2) and against one or two lineages of influenza B viruses. Because of the rapid antigenic evolution of the surface proteins, primarily the hemagglutinin, the vaccine requires regular updates to remain effective. The World Health Organization (WHO) maintains a global surveillance system and collects and analyzes data biannually to recommend strains included in the vaccine for the next influenza seasons. Because of the lengthy vaccine production process, this update has to be performed one year in advance. This is the “vaccine strain prediction problem”, in which predicting a strain representing the predominant antigenic virus type has to be made one year ahead of time. A false or late prediction results in vaccines with suboptimal protection and an increase in influenza-related cases and mortality [32].

Over the last 10 years, we have worked on computational methods for predicting a necessary vaccine strain, because of the emergence of a novel antigenic type, and investigated which strains could be used for this update. In [33], we describe allele-dynamics plots, which rank alleles, corresponding to sets of amino acid changes, in descending order by their increase in frequency over consecutive seasons. In a constantly sized, homogenous population, this, with some statistical variance, relates directly to the strength of the selective advantage that they may provide; “they may” because they may also all be neutral. An allele is defined as the set of changes on an individual branch within a genealogy, which allows for consideration of the genetic context of individual changes and for distinguishing between events when individual changes are introduced multiple times. This method accurately ranked the alleles with amino acid changes of HA corresponding to the changes between successive antigenic types of influenza A/H3N2 viruses over the analyzed time period. Thus in practice, it appears that the antigenic evolution of human influenza A viruses, likely as a result of the rapid global movements of their human hosts, may be represented by such a simple population genetics model.

AD-plots, however, do not give us information about the antigenicity-altering nature of the respective alleles; thus they do not directly point to antigenically altered alleles rising to predominance. We thus devised antigenic trees, or more generally, phenotype trees [34], a method for regressing antigenic distance measurements between viruses onto a tree. This allowed us to estimate the antigenicity-altering effect of alleles and, from that, of individual sites or amino acid changes, in agreement with experimental studies. Putting together AD-plots and antigenic trees allowed for predicting suitable vaccine strains with improved performance in retrospective testing, namely, in several cases one season earlier than the WHO [35].

Differently from HA sequence data, which is made public in a timely manner [36], only little antigenic data is released, which prevents providing predictions with this vaccine strain prediction procedure in practice. We thus investigated whether we could identify some general characteristics of the antigenic evolution of influenza A/H3N2 viruses that would be helpful for predicting vaccine strain updates. To this end, we derived antigenicity-altering values per site from the antigenic-allele information and used a graph-cut algorithm for finding antigenicity-altering sites on the HA structure that cluster together, resulting in six patches of sites identified for HA [37]. This removed several spurious antigenicity-altering sites reported with the antigenic tree because of co-localization on a branch with a truly antigenicity-altering site. Of the six patches, patches 1 and 2 surrounded the receptor binding site of HA. Almost all transitions between consecutive antigenic variants for H3N2 viruses included a change in either one or the other, as well as for time periods following the time period covered by the antigenic data used for inference.

**Figure 4:**
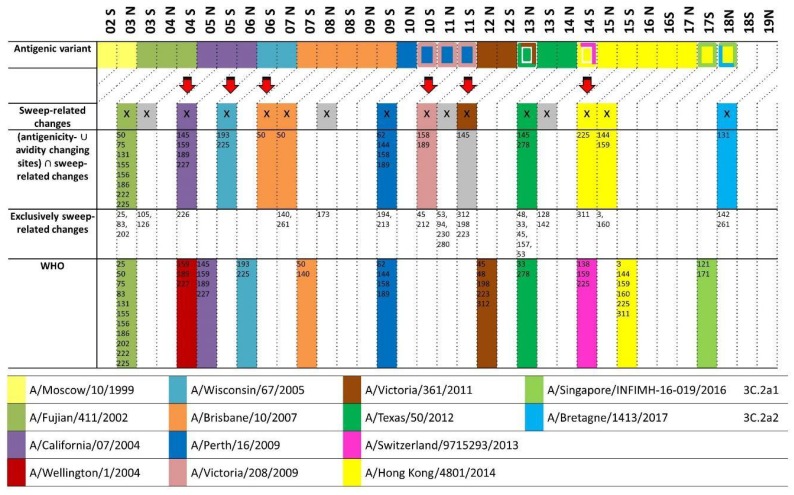
Comparison of predominant antigenic types for human influenza A/H3N2, predictions using sweep dynamics (SD) plots and recommendations made by the World Health Organization (WHO). The selection of a vaccine strain takes place two seasons before the vaccine is available. Any prediction of newly arising antigenically novel strains should therefore be compared to the predominant antigenic type two seasons later (indicated by diagonal lines in plot). (**First row**) Colored boxes indicate the predominant antigenic variant, and additional colored borders indicate different dominantly circulating strains matching the same antigenic variant; (**Second row**) For the SD plots’ analysis, seasons are marked with an “X” if sweep-related changes distinguish the WHO-selected vaccine strain from the previous strain. Seasons with sweep changes not associated with antigenicity-altering or avidity-changing sites are marked in grey; (**Third row**) Sweep-related changes in antigenicity-altering or avidity-changing sites. Using these as a criterion for vaccine strain updates results in a simultaneous or earlier detection of newly emerging antigenic types than with the procedure utilized by the WHO; (**Fourth row**) Detected sweep-related sites known to change neither the avidity nor the antigenicity; (**Fifth row**) WHO recommendations. Until 2017N, performance was evaluated by retrospective testing, in which data from after the time of the WHO vaccine strain meeting for a particular season was excluded from the analysis. From 2017S onwards, predictions were made for the future and can be monitored live at https://github.com/hzi-bifo/SDplots_VaccineUpdates.

We added a statistical significance measure to the AD-plots for identifying alleles—now corresponding to individual amino acid changes on a specific branch—rising to predominance. These “sweep dynamics” (SD) plots indicate alleles increasing significantly in frequency over consecutive seasons and with abundances above 50%—thus “sweeping” towards predominance. This, together with antigenic patch information, provided the basis for a computational vaccine strain prediction procedure (Figure 4). We observed good performance and earlier detection of novel emerging antigenic variants, without analyzing antigenic data from after 2003 [38]. Since September 2017, we have provided predictions simultaneously to the WHO (Figure 4). Currently, this method predicts a necessary update of the H3N2 vaccine strain with a member of the 3C.2a2 clade, while the WHO has not (yet) recommended this change. In several years, a performance comparison under realistic circumstances will be possible. This work was done by Alice C. McHardy, Lars Steinbrück, Christina Kratsch, Thorsten Klingen, and Susanne Reimering.

### 2.12. Mark Zwart Talked about Evolutionary Perspectives on the Role of Copy Number Variation in Viral and Bacterial Adaptation

In many organisms, multiple copies of parts of the genome can be present, and the number of copies present can vary within populations. We refer to such genomic diversity as copy number variation (CNV). In bacteria, CNV plays an important role in adaptation, for example, in the presence of antibiotics. Although the beneficial effects of CNV are often small, the mutation supply for CNV is very large. Given the amount of standing genetic variation present in most bacterial populations, selection can immediately act on CNV. CNV is therefore typically the first line of bacterial genetic adaptation and is often followed later by single-nucleotide variants with greater beneficial effects and a reversion of CNV. Although CNV plays an important role in bacterial adaptation, we know very little about the role of CNV in virus adaptation. Virus genomes tend to be highly streamlined: any inserted sequences—whether duplications of virus genes or heterologous genes—typically are rapidly purged. One might therefore expect that CNV does not play a role in virus adaptation, but surprisingly there are examples to the contrary. CNV is known to play a transient role in poxvirus adaptation to new hosts: influenza A virus can downregulate the frequency of the genome segment coding for its neuraminidase, and baculoviruses can regulate expression of infectivity factors through polymorphic genomic deletions. Multipartite (or multi-component) viruses take a more radical approach and package their different genome segments into different virus particles. At least one virus particle containing each genome segment must then reach a new cell for the virus to be successfully transmitted. This strange genome and virus particle organization comes at a huge cost: a predicted inefficient transmission of the virus, both within and between hosts. Why has strange genome organization arisen? Here, we explored this issue for a tripartite plant RNA virus, alfalfa mosaic virus. First, we revisited classical virological work and considered infection kinetics in detail, documenting that multipartition does indeed impose a huge cost on virus infectivity. Second, we found that the different genome segments are not present at the same frequency, similar to observations with nanoviruses. When perturbed, the frequency of these segments converges to a stable equilibrium, which is host-species dependent. These observations suggest that changes in the frequency of genome segments might be a strategy for adapting to different environments, for example, because they lead to differences in the expression of viral genes. Third, models of virus evolution show the importance of genetic bottlenecks for adaptation by changes in the frequency of genome segments. These bottlenecks generate the variation in segment frequencies on which selection can then act, which allows for rapid, “mutation-free” adaptation to different environments. Finally, we used these models of virus evolution to consider how heterogeneous environments must be to favor a multipartite over a monopartite genome organization. Changes in genome segment frequencies might therefore play a similar role to CNV in other viruses and bacteria, although the lack of linkage between these copies imposes both unique costs to infectivity and a very large, continuous supply of variation for natural selection to act on. These results stress that CNV can sometimes play an important role in virus adaptation and suggests that it be given due consideration when considering high-throughput sequencing data.

### 2.13. Susana Posada Céspedes Spoke on the Bioinformatics Pipeline V-pipe for Inferring Viral Diversity from High-Throughput Sequencing Data

RNA viruses are responsible for various infectious diseases in humans, animals, and crops, representing a burden to human health as well as causing substantial economic losses. One major limitation when treating viral infections is the great genetic diversity featured by RNA viruses. The existence of a heterogeneous mixture of viral strains has implications on viral pathogenesis, virulence, and disease progression. HTS technologies have opened up new possibilities for in-depth characterization of the diversity of viral samples, and the ultimate goal is to define standards for research as well as to incorporate the use of this technology into clinical diagnosis. In order to support HTS-based viral genomics, we devised and implemented a bioinformatics pipeline, V-pipe. The workflow integrates tools for the assessment of the viral diversity at the levels of both single nucleotide variants (SNVs) and viral haplotypes. V-pipe is an automated and easy-to-use framework delivering reproducible results. Furthermore, it features a modular and extensible architecture that enables developers to easily test their own tools, contributing towards the establishment of best practices for clinical diagnostics. This work was done by Susana Posada-Céspedes, David Seifert, Tobias Marschall, and Niko Beerenwinkel.

### 2.14. Philippe Lemey Described Bayesian Phylogenetic and Phylodynamic Data Integration Using BEAST

The field of phylodynamics has witnessed a rich development of statistical inference tools with increasing levels of sophistication, but these tools have traditionally focused on sequences as their sole data source. Integrating various sources of information with genomic data promises to deliver more precise insights into infectious diseases and to increase opportunities for statistical hypothesis testing.

Emerging concepts of data integration are now stimulating new advances in Bayesian evolutionary inference methodology. We have worked on such methodology in the BEAST software, which represents a coherent statistical framework for integrated analyses of sequences and traits using Bayesian inference techniques. These approaches were applied in various outbreak scenarios and real-time genomic epidemiology. For discrete traits, we have developed generalized linear modeling extensions of continuous-time Markov chain processes that allow the integrating and testing of covariates. Applications using this approach have focused on the dynamics of spatial spread for viruses such as influenza and Ebola. We have also developed equivalent random walk models for continuous traits to test specific hypotheses about the impact of border closures on Ebola dispersal frequency. The 2013–2016 West African Ebola epidemic marked the start of real-time genomic sequencing, and this methodology can make an important impact on outbreak responses. Recent work during the Lassa fever virus outbreak demonstrated this and brings about new bioinformatics challenges.

## 3. Poster Sessions

One important facet of the annual EVBC meeting was the poster sessions. The friendly atmosphere was instrumental to developing fruitful discussions during the long poster sessions combined with lunches and dinners.

## 4. Conclusions

The second EVBC meeting was a success in its primary aims of bringing virologists and bioinformaticians together to share ideas, agree on differences of opinion, and create a future action agenda.

Collectively, this meeting hosted 15 oral presentations and 2 long poster sessions over a two day period with around 120 participants. This report presents an overview of salient new developments and novel research findings that emerged during the second EVBC annual meeting. We hope that the highlights provided in this report will encourage interested researchers to join us at the Third Annual Meeting of the European Virus Bioinformatics Center, which will be held in 2019 at the University of Glasgow; wish to see you all again.

## 5. Affiliations of Attendees

Name Affiliation City-CountryAkbar Adjie PratamaUniversity of GroningenGroningen-NetherlandsAlessandro RossiUtrecht UniversityUtrecht-NetherlandsAlexander SchoenhuthCentrum Wiskunde & Informatica Amsterdam-NetherlandsAlexander KurilshikovUniversity Medical Center GroningenGroningen-NetherlandsAlexandra BelanovaFree University of BerlinBerlin-GermanyAlexandra ZhernakovaUniversity Medical Centre GroningeGroningen-NetherlandsAli ElbeheryHelmholtz Center MunichMunich-GermanyAlice FusaroIstituto Zooprofilattico Sperimentale delle VenezieLegnaro-ItalyAlice McHardyHelmholtz Center for Infection ResearchBraunschweig-GermanyAna Rita CostaDelft University of TechnologyDelft-NetherlandsAnita SchurchUniversity Medical Center UtrechtUtrecht-NetherlandsAnne KupczokKiel UniversityKiel-GermanyAnnelies KronemanNetherlands National Institute for Public Health and the EnvironmentBilthoven-NetherlandsArno C. AndewegErasmus Medical CenterRotterdam-NetherlandsArthur GruberUniversity of São PauloSão Paulo-BrazilArthur EdridgeUniversity of AmsterdamAmsterdam-NetherlandsAvraam TapinosUniversity of ManchesterManchester-UKBas Oude MunninkErasmus University Medical CenterRotterdam-NetherlandsBas E. DutilhUtrecht UniversityUtrecht-NetherlandsBashar IbrahimFriedrich Schiller University JenaJena-GermanyBastiaan Von MeijenfeldtUniversity of UtrechtUtrecht-NetherlandsBen StampMRC-University of Glasgow Centre for Virus ResearchGlasgow-ScotlandBrett PickettJ. Craig Venter InstituteRockville-USACeren SimsekKatholieke Universiteit LeuvenLeuven-BelgiumCesare GruberNational Institute for Infectious Diseases Rome-ItalyChenyan ShiKatholieke Universiteit LeuvenLeuven-BelgiumCormac KinsellaUniversity of AmsterdamAmsterdam-NetherlandsC. Aparicio-MaldonadoDelft University of TechnologyDelft-NetherlandsDaniel GarzaCMBINijemegen-NetherlandsDaniela BezemerHIV Monitoring FoundationAmsterdam-NetherlandsDavid RobertsonMRC-University of Glasgow Centre for Virus ResearchGlasgow-ScotlandDavid NieuwenhuijseErasmus University Medical CenterRotterdam-NetherlandsDaniel DesiroFriedrich Schiller University JenaJena-GermanyDennis SchmitzNetherlands National Institute for Public Health and the EnvironmentUtrecht-NetherlandsDino McMahonFree University of BerlinBerlin-GermanyDivyae PrasadUtrecht UniversityUtrecht-NetherlandsEduardo De EsesarteUtrecht UniversityUtrecht-NetherlandsErik De VriesUtrecht UniversityUtrecht-NetherlandsEric HesterRadboud UniversityNijmegen-NetherlandsEugene KooninNational Center for Biotechnology InformationBethesda-USAEva WeissUniversity of YorkYork-UKFrancesca YoungMRC-University of Glasgow Centre for Virus ResearchGlasgow-UKFrançois EnaultUniversité Clermont AuvergneClermont Ferrand-FranceFrank Van KuppeveldUtrecht UniversityUtrecht-NetherlandsFranklin NobregaDelft University of TechnologyDelft-NetherlandsGianpiero ZamperinIstituto Zooprofilattico Sperimentale delle VenezieLegnaro-ItalyGonçalo PiedadeRoyal Netherlands Institute of Sea Research’t Horntje-NetherlandsGuillaume BeauclairInstitutPasteurParis-FranceHarry VennemaNetherlands National Institute for Public Health and the EnvironmentBilthoven-NetherlandsHilde HerremaUniversity of AmsterdamAmsterdam-NetherlandsHimanshu ManchandaUniversity of GreifswaldGreifswald-GermanyJelle MatthijnssensKatholieke Universiteit LeuvenLeuven-BelgiumJelmer FaberRadboud UniversityNijmegen-NetherlandsJenna KellyInstitute of Virology and ImmunologyBern-SwitzerlandJoan Martí-Carreras Katholieke Universiteit LeuvenLeuven-BelgiumJoanna KaczorowskaUniversity of AmsterdamAmsterdam-NetherlandsJoseph BusbyMRC-University of Glasgow Centre for Virus ResearchGlasgow-UKJulian SusatKiel UniversityKiel-GermanyKaat RamaekersRega Institute for Medical ResearchLeuven-BelgiumKathrin TrappeRobert Koch-InstituteBerlin-GermanyKevin LamkiewiczFriedrich Schiller University JenaJena-GermanyKim NgStatens Serum InstituteCopenhagen-DenmarkKoen DeforcheEmweb Genome DetectiveHerent-BelgiumKoenraad Van DoorslaerUniversity of ArizonaTucson-United StatesKsenia ArkhipovaUtrecht UniversityUtrecht-NetherlandsLeen BellerKatholieke Universiteit LeuvenLeuven-BelgiumLi DengHelmholtz Centre MunichMunich-GermanyLia Van Der HoekUniversity of AmsterdamAmsterdam-NetherlandsLisa FalkAmsterdam Medical CenterAmsterdam-NetherlandsLore Van Espen Katholieke Universiteit LeuvenLeuven-BelgiumLukasz RabalskiUniversity of GdanskGdansk-PolskaMartin DeijsUniversity of AmsterdamAmsterdam-NetherlandsMartin HölzerFriedrich Schiller University JenaJena-GermanyMaike HerrmannPaul-Ehrlich-InstituteLangen-GermanyManja MarzFriedrich Schiller University JenaJena-GermanyMareike DörrPaul-Ehrlich-InstituteLangen-DeutschlandMargo SchullerUtrecht UniversityUtrecht-NetherlandsMarijn Thijssen Katholieke Universiteit LeuvenLeuven-BelgiumMaria Ines GismondiNational Institute for Agriculture ResearchBuenos Aires-ArgentinaMark ZwartNetherlands Institute for EcologyWageningen-NetherlandsMarkus UlrichRobert Koch InstituteBerlin-GermanyMatthew CottenErasmus University Medical CenterRotterdam-NetherlandsMengmeng FanRega InstituteLeuven-BelgiumMichael HuberUniversity of ZurichZurich-SwitzerlandMichael WolfingerMedical University of ViennaVienna-AustriaMichel Christoph KochUniversity of BernBern-SwitzerlandMy PhanErasmus University Medical CenterRotterdam-NetherlandsOsvaldo ZagordiUniversity of ZurichSwitzerlandPhilippe Le MercierSwiss Institute of BioinformaticsGeneva-SwitzerlandPhilippe LemeyKatholieke Universiteit LeuvenLeuven-BelgiumRebecca HalbachRadboud University Medical Center Nijmegen-NetherlandsRichard ScheuermannJ. Craig Venter InstituteLa Jolla-USARobert GiffordMRC-University of Glasgow Centre for Virus ResearchGlasgow-UKRonald DijkmanInstitute of Virology and ImmunologyBern-SwitzerlandRonald Van RijRadboud University Medical CenterNijmegen-NetherlandsSam NooijNetherlands National Institute for Public Health and the EnvironmentBilthoven-NetherlandsSander Van BoheemenErasmus University Medical CenterRotterdam-NetherlandsSanzhima GarmaevaUniversity of GroningenGroningen-NetherlandsS. Calvignac-SpencerRobert Koch InstituteBerlin-GermanyShivaprasad PatilKatholieke Universiteit LeuvenLeuven-BelgiumStan BrounsDelft University of TechnologyDelft-NetherlandsSusana Posada CéspedesETH ZurichBasel-SwitzerlandSyriam Na AyudhyaErasmus University Medical CenterRotterdam-NetherlandsThierry JanssensNetherlands National Institute for Public Health and the EnvironmentBilthoven-NetherlandsThor JohannesenStatens Serum InstituteCopenhagen-DenmarkVerena KufnerUniversity of ZurichZurich-SwitzerlandWannisa RitmahanVrije UniversiteitUtrecht-NetherlandsWard DeboutteKatholieke UniversiteitLeuven-BelgiumWim DumonCreating Innovative SoftwareLeuven-BelgiumYifan ZhuWageningne UniversityWageningen-Netherlands
